# Differences in the diagnosis and management of pyriform sinus fistula between newborns and children

**DOI:** 10.1038/s41598-019-55050-9

**Published:** 2019-12-06

**Authors:** Qingfeng Sheng, Zhibao Lv, Weijue Xu, Jiangbin Liu

**Affiliations:** Department of General Surgery, Shanghai Children’s Hospital, Shanghai Jiao Tong University, Shanghai, 200062 China

**Keywords:** Outcomes research, Paediatric research

## Abstract

Pyriform sinus fistula (PSF) is a rare congenital entity. We hypothesized that clinical features, diagnosis and treatment may differ between newborns and children. 190 patients diagnosed with PSF were divided into two groups: neonatal (n = 15) and childhood (n = 175). The medical records including demographic and clinical data were retrospectively analyzed. There were 102 boys and 88 girls in this study. Most patients presented as a left-sided neck lesion. A neck mass, with or without infection and respiratory distress, was the common finding in newborn patients. Prenatal diagnosis was made in three cases. However, in childhood group, initial symptoms were neck abscess (78.8%), acute thyroiditis (11.4%), neck mass (6.9%), and thyroid lesion (2.8%). The presence of a cervical mass with air pocket showed on CT image was thought to be the pathognomonic finding of neonatal PSF. The diagnosis was usually established by barium esophagography in older children. Delayed accurate diagnosis was detected in both groups. The median time from onset to diagnosis was 22 months and 1 year respectively. Endoscopic-assisted open surgery was performed successfully in all patients, with good outcomes in majority cases of both groups (93.3% and 95.4%). Recurrence was developed in 5 patients. PSF should be suspected in newborns with cervical mass and in children with recurrent neck infection, especially on the left side. Early diagnosis and treatment might avoid repeated surgical procedures. Complete resection of the cyst and fistula in non-infected state is essential for good outcomes.

## Introduction

Pyriform sinus fistula (PSF) is a rare congenital entity, originating from the failure obliteration of the third or fourth pharyngeal pouches^1,2^. Most PSF occurs in older children and presents with repeated neck infection or mass. A few prenatal and neonatal cases have been reported in the English literature^3–6^. In neonates, PSF presents with a cervical cystic lesion resulting in compressive symptoms or infection. We hypothesized that clinical features, diagnosis and treatment may differ between newborns and children.

## Material and Methods

The medical records of 190 cases diagnosed with PSF at our institution during January 2010 until March 2018 were retrospectively reviewed. The exclusion criteria: patients who had not yet undergone definitive surgery, who had incomplete data, withdrawal of treatment, age ≧ 18 years. PSF, which is generally considered as a congenital anomaly, can present both in newborns and in children. According to the age of initial clinical presentation, patients were classified into two groups: neonatal (n = 15) and childhood (n = 175). The medical records including demographics, clinical findings, imaging, treatment and outcomes were analyzed. All the endoscopic-assisted operations were performed by chief surgeon (Z. Lv). The details of surgical procedures have been described previously by our group^2,7^. The current study was carried out in accordance with The Code of Ethics of the World Medical Association (Declaration of Helsinki). Ethical approval was obtained from the Ethics Boards of Shanghai Children’s Hospital (#2019R017). Written informed consents (for both study participation and publication of identifying information/images in an online open-access publication) were obtained from parents and/or legal guardians on behalf of the children. Follow-up by phone or interview in our clinics was conducted by an experienced surgeon (Q. Sheng). Statistical analysis in this study was performed by the SPSS 17.0 software (Chicago, IL, United States). *χ*^2^ test or Fisher exact test was used to compare frequencies. Difference was considered to be significant when P < 0.05 (α = 0.05, two-tailed).

## Results

### Clinical features

**Figure 1:**
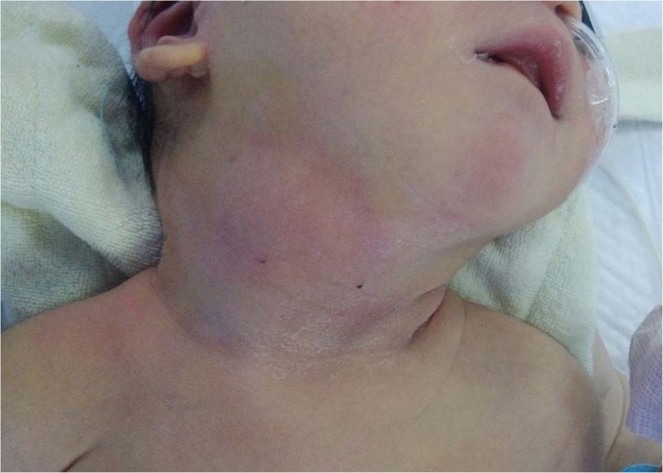
A cervical mass on the right side in a 2-day-old male newborn.

**Figure 2:**
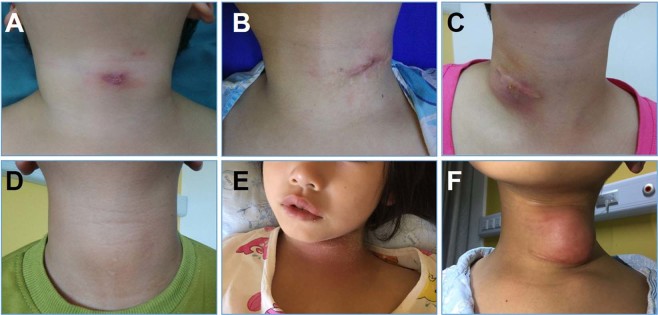
Clinical findings of PSF in older children. Depiction of neck after repeated incision and drainage (**A**–**C**), no infection (**D**), acute infection (**E**), and recurrence after traditional open surgery (**F**).

The demographic and clinical data were shown in Table 1. There were 102 boys and 88 girls in this study (male/female ratio, 1.16:1). No gender predominance was observed between neonatal and childhood groups (P = 0.789). In the neonatal group, the median age at presentation and diagnosis was 1 day (range, 0–10 days) and 22 months (range, 5 days – 11 years) respectively. In the childhood group, the median age at presentation and diagnosis was 3.5 years and 6 years. Most patients presented as a left side lesion (93.3% in neonatal, 93.1% in childhood, P = 0.669). All neonatal patients presented with a neck mass with or without infection (Fig. 1). Three cases developed respiratory distress due to lesion compression, requiring incisional drainage or endotracheal intubation. Cervical mass was detected prenatally in three patients (3/15, 20%). No ex utero intrapartum treatment (EXIT) procedure was needed. However, in childhood group (Fig. 2), the clinical presentations were neck abscess (n = 138, 78.8%), acute suppurative thyroiditis or thyroid abscess (n = 20, 11.4%), neck mass with or without dyspnea (n = 12, 6.9%), and thyroid nodule (n = 5, 2.8%), which were in consistent with previous studies^8–10^.

**Table 1:** Clinical features of 190 cases with pyriform sinus fistula.

Features	Neonatal (n = 15)	Childhood (n = 175)	P value
Male, n (%)	9 (60%)	93 (53.1%)	0.789
Age at presentation, median (range)	1 day (0–10 days)	3.5 years (10 months-12 years)	NA
Age at diagnosis, median (range)	22 months (5 days-11 years)	6 years (1 year-15 years)	NA
Time from presentation to diagnosis, median (range)	22 months (4 days-11 years)	1 year (0–12 years)	NA
Side of lesion, n (%)			0.669
Left	14 (93.3%)	163 (93.1%)	
Right	1 (6.7%)	9 (5.1%)	
Bilateral	0	3 (1.7%)	
Initial presentations, n (%)			<0.001
Neck mass	8 (53.3%)	11 (6.3%)	
Neck mass with dyspnea	3 (20%)	1 (0.6%)	
Neck mass with infection or neck abscess	4 (26.7%)	138 (78.8%)	
AST/thyroid abscess	0	20 (11.4%)	
Thyroid nodule	0	5 (2.8%)	
Follow-up, median (range)	1.5 years (1 year-4 years)	3 years (10 months-8 years)	NA

NA, not applicable.

### Diagnostic investigations

**Figure 3:**
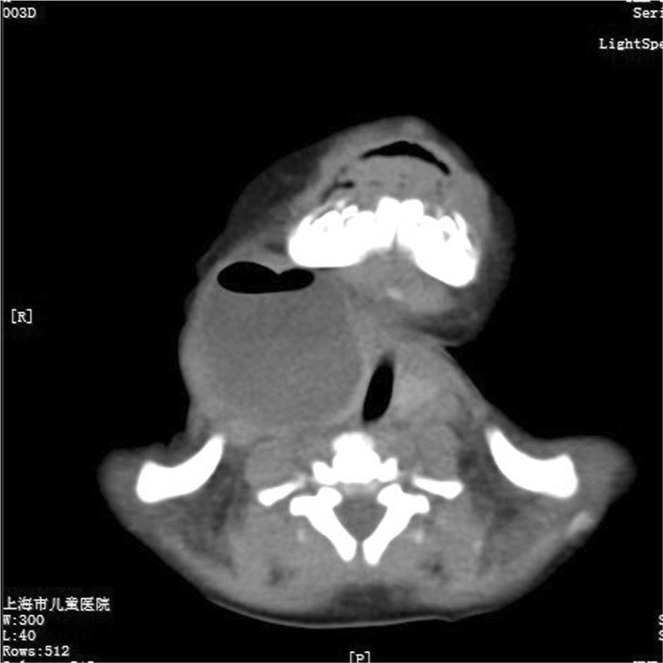
Axial computed tomography scan shows an air-filled cyst with deviation of the trachea (the same patient in Fig. 1).

**Figure 4:**
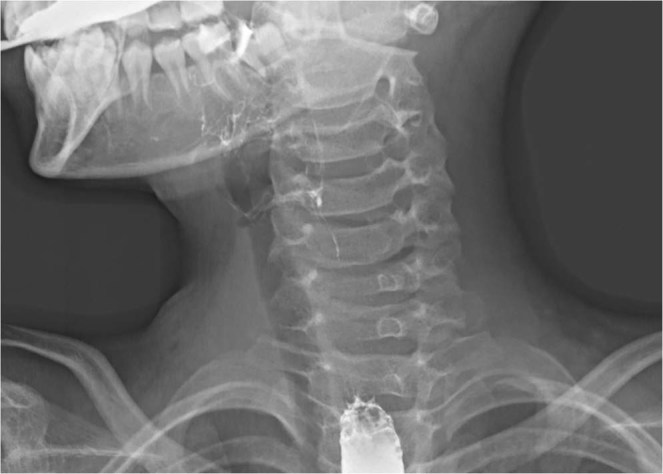
Barium esophagogram shows the left-sided fistula tract in a 9-year-old boy.

In the neonatal series, 12 patients were referred from outside hospitals. Ultrasonography combined with computed tomographic (CT) scan showed a simple cystic lesion containing air pocket, with deviation of the trachea (Fig. 3). Barium esophagography was performed in 2 patients in the neonatal period. Unfortunately, no PSF was delineated in any cases. On the contrary, all patients in the childhood group underwent barium esophagraphy with an excellent positive predictive value (PPV, 100%, Fig. 4). Oral-contrast or intravenous-contrast enhanced CT also had satisfactory PPV. Delayed accurate diagnosis was detected in both groups (the median time from presentation to diagnosis is 22 months and 1 year respectively).

### Treatments and outcomes

The summary of treatment was shown in Table 2. Previous treatment in the neonatal group were incision and drainage (I&D) in 12 cases, traditional open surgery in 2 cases, and endoscopic-assisted open surgery in one case (the fistula was successfully located with the assistance of endoscopy, cyst and fistula were then completely excised). The number of I&D attempts ranged from 1 to 6 (median, 2). Three patients received traditional open surgery as primary treatment or after the failure of I&D (all from outside institutions), but neck infection recurred.

**Table 2:** Summary of treatments and outcomes.

Features	Neonatal (n = 15)	Childhood (n = 175)	P value
Previous treatments			0.011
Only antibiotics before endoscopic assisted operation, n (%)	0	32 (18.3%)	
I&D as initial procedure, n (%)	12 (80%)	133 (76%)	
I&D in neonatal period	7	NA	
No. of I&D attempts, median (range)	2 (1–6)	2 (1–12)	
Traditional open surgery as initial procedure, n (%)	2 (13.3%)	10 (5.7%)	
Endoscopic-assisted operation, n (%)	1 (6.7%)	0	
Age at endoscopic-assisted operation, median (range)	2 years (9 days-13 years)	6 years (1 year-17 years)	NA
**Outcomes**			
Complications, n (%)	^a^1 (6.7%)	^b^8 (4.6%)	0.531
Recurrence, n (%)	0	5 (2.9%)	0.660

NA, not applicable; I&D, incision and drainage.

^a^One patient developed wound infection.

^b^Eight patients developed postoperative complications: wound infection (1), temporary vocal fold motion impairment (1), Horner syndrome (1), recurrence (5).

Among the childhood group, before endoscopic-assisted open surgery, initial treatments included I&D in 133 cases (76%), traditional open-neck in 10 cases (5.7%), and conservative therapy in 32 cases (18.3%). Repeated I&D after failed previous procedures (I&D or open surgery) was performed in 91 patients. Traditional open surgery or laser cauterization, both as initial treatment or after the failure of I&D, was performed in 30 patients, all resulting in recurrence.

All 190 cases underwent endoscopic-assisted open surgery in our institution. Good outcome was achieved in both groups (93.3% vs 95.4%, P = 0.531, Table 2). One infant in the neonatal group developed wound infection. And eight patients in the childhood group demonstrated postoperative complications during a median follow-up of 3 years (range, 10 months – 8 years). Recurrence was developed in 5 cases (2.9%), 3 of them were treated by re-excision. The other 2 patients were managed conservatively with antibiotics intravenously and waiting for definitive operation. In terms of recurrence, no statistical significance was detected between these two groups (P = 0.66).

## Discussion

Pyriform sinus fistula in pediatric patients is recognized as congenital origin. Analysis of the results of the current study shows two clinical form of PSF: neonatal and childhood. There are differences in clinical manifestation, diagnostic method and treatment between these two groups. Clinical presentations of PSF vary with age^11–13^. A neck cyst, may or may not be infected and compressing surrounding structure, is common clinical findings among neonatal patients. In contrast, PSF in older children presents with repeated neck abscess. Multiple I&D and open surgery might result in an external fistula formation. Recently, prenatal diagnosis of pyriform sinus fistula has been made based on the findings of ultrasonography and magnetic resonance imaging (MRI). And EXIT procedure was applied in some cases^5,6,14^. PSF should be suspected in newborns with a cervical mass and in children with recurrent neck infection, especially on the left side.

The diagnosis of PSF in older children is usually established by barium esophagopraphy and CT in our series. However, barium swallow study has limited values in newborns. CT or MRI are the preferred methods for neonates. The presence of a cervical mass with air pocket is thought to be the pathognomonic finding of neonatal PSF. Misdiagnosis is common in both neonatal and childhood groups. Only three cases admitted to our hospital in the neonatal period were considered the diagnosis of PSF. Other 12 patients in the neonatal group were misdiagnosed as lymphangioma, lymphadenopathy, and even thyroglossal duct cyst. Preoperative suspicion of PSF facilitates early accurate diagnosis and avoiding repeated surgical procedures (e.g. I&D).

Turning to the issue of treatment, identification of the fistula tract is the key point during the operation. Intraoperative endoscopy will help to visualize the internal opening of pyriform sinus. From our experience, it is safe to perform intraoperative endoscopy even in newborns. Failure to excise the entire fistula tract can always lead to recurrence. Because of the close relationship between the cyst/fistula and thyroid, it is reasonable to perform partial thyroidectomy if needed. The recurrence rate in current study is low (0% and 2.9%, respectively). As in our and other investigators’ reports^2,6,7,11^, complete surgical excision in non-infected state is once again recommended, but great care must be taken because of the proximity of the sympathetic nerves and the recurrent laryngeal nerves. Early diagnosis, entire resection with the help of endoscopy might translate into less morbidity and better outcomes.

Recent studies suggested that obliteration of the inner orifice is supposed to be an effective method for the management of PSF. Different modalities were used to seal the inner opening, including electrocauterization^15,16^, chemocauterization^17,18^, laser coagulation^11,19,20^, and biocauterization^21,22^. However, recurrence is inevitable in some cases because of incomplete obliteration of the tract opening and/or the remaining of the epithelial lining of the fistula tract. Wang *et al*.^20^ reported that the success rate was 55.9% after first laser cauterization. Nearly half patients required two or more treatments. It seems reasonable to perform complete resection of the fistula when multiple recurrences occur.

We have the following limitations: single center’s experience, retrospective design, few neonatal cases, limited experience of endoscopic-assisted open surgery in newborns, lacking long-term outcomes (especially in neonatal group).

## Conclusion

Clinical presentations of PSF vary with age. Most cases are left-sided. Delayed diagnosis is common in both neonatal and childhood groups. CT scan showed the characteristic air-filled cyst in neonatal PSF. However, the diagnosis was usually established by barium esophagography in older children. Early diagnosis and treatment might avoid repeated surgical procedures. Complete resection of the cyst and fistula in noninfected state is essential for good outcomes.
